# Kawasaki disease in infants less than one year of age: an Italian cohort from a single center

**DOI:** 10.1186/s12887-019-1695-0

**Published:** 2019-09-07

**Authors:** Greta Mastrangelo, Rolando Cimaz, Giovani Battista Calabri, Gabriele Simonini, Donatella Lasagni, Massimo Resti, Sandra Trapani

**Affiliations:** 1 Pediatric Residency program, Meyer Children’s Hospital, University of Florence, Florence, Italy; 2 Department of Clinical Sciences and Community Health, University of Milan, Milan, Italy; 3 Cardiology Unit, Meyer Children’s Hospital, University of Florence, Florence, Italy; 4 Rheumatology Unit, Meyer Children’s Hospital, University of Florence, Florence, Italy; 5 Department of Pediatrics, Meyer Children’s Hospital, University of Florence, Florence, Italy

**Keywords:** Kawasaki disease, Infant, Coronary artery aneurysms, AST, Caucasians

## Abstract

**Background and aims:**

Few data are currently available for Kawasaki disease (KD) below 12 months especially in Caucasians. This study aims to analyze clinical and laboratory features of KD among an Italian cohort of infants.

**Methods:**

A retrospective chart review of KD children aged less than 1 year at time of disease onset between January 2008–December 2017 was performed. Clinical data, laboratory parameters, instrumental findings, treatment and outcome were collected in a customized database.

**Results:**

Among 113 KD patients, 32 (28.3%) were younger than 1 year. Nineteen patients aged below 6 months, and three below 3 months. The median age was 5.7 ± 2.7 months. The mean time to diagnosis was 7 ± 3 days and was longer in the incomplete forms (8 ± 4 vs 6 ± 1 days). Conjunctival injection was present in 26 patients (81.2%); rash in 25 (78.1%); extremity changes in 18 (56.2%); mucosal changes in 13 (40.6%,) and lymphadenopathy only in 7 (21.8%). Mucosal changes were the least common features in incomplete forms (18.2%). Twenty-two patients (68.7%) had incomplete KD. Nineteen (59.4%) had cardiac involvement, of whom 13 (59.0%) had incomplete form. ESR, PCR and platelet values were higher in complete KD; especially, ESR resulted significantly higher in complete forms (80 ± 25.7 mm/h vs 50 ± 28.6 mm/h; *p* = 0.01). Conversely, AST level was statistically significant higher in patients with incomplete forms (95.4 ± 132.7 UI/L vs 29.8 ± 13.2 UI/L; *p* = 0.03). All patients received IVIG. Response was reported in 26/32 patients; 6 cases needed a second dose of IVIG and one required a dose of anakinra.

**Conclusion:**

In our cohort, incomplete disease was commonly found, resulting in delayed diagnoses and poor cardiac prognosis. Infants with incomplete KD seem to have a more severe disease and a greater predilection for coronary involvement than those with complete KD. AST was significantly higher in incomplete forms, thus AST levels might be a new finding in incomplete forms’ diagnosis. Eventually, we highlight a higher resistance to IVIG treatment. To our knowledge this is the first study involving an Italian cohort of patients with KD below 12 months.

## Background

Kawasaki disease (KD) is an acute medium vessel vasculitis of childhood, typically involving coronary arteries [1–3]. It is one of the most common pediatric vasculitis and the commonest cause of acquired heart disease in children in developed countries. Current annual incidence rates of KD in Japan, Korea and Taiwan are 264.8, 134.4 and 69 cases per 100,000 children below 5 years, respectively [4–6]. The incidence rate in Italy is about 5.7 per 100,000 children 0–14 years old, and 14.7 for children younger than 5 years [7]. It is seldom reported below 3 months of age: only 1.6% of all KD patients [8]. Current literature reports that infants below 12 months of age have a higher prevalence of incomplete and atypical KD (40%) compared to older patients (10–12%) [1]. Delayed diagnosis and treatment, higher incidence of coronary arteries abnormalities, and greater intravenous immunoglobulin (IVIG) resistance frequently occur in KD infants [9–16]. There is paucity of literature exclusively on KD below the age of 12 months and little information is available on this aspect of KD from any developed country [16]. To the best of our knowledge there are no published data on KD in infancy from other Italian centers. We report the clinical data, laboratory profile, instrumental findings and management of 32 children with KD, aged below 12 months who were admitted to our institution in the last 10 years. Furthermore, within such cohort we compared babies with complete KD to those with incomplete form, in order to identify any clinical or laboratory data, potentially useful in early detecting the incomplete forms. Moreover, babies with coronary involvement were compared with those without cardiac impairment, in an effort to detect any clinical or hematological feature, which could predict cardiac involvement.

## Methods

This is a retrospective chart review of children diagnosed with KD (ICD-9 discharge code 4461) between January 1st, 2008 and December 31st, 2017. KD children younger than 12 months of age at disease onset were selected and included in the analysis. Demographic (age, gender) and clinical data, including season of onset, time to diagnosis, signs and symptoms, cardiac involvement, treatment and outcome were collected. Delayed diagnosis was defined as a KD diagnosis made after day 10 of illness – the cut-off period generally considered most appropriate for administration of IVIG. Complete and incomplete KD were defined according to the American Heart Association definition [3]. Echocardiograms were performed at diagnosis, 2 weeks after disease onset and after 6–8 weeks in all patients. Additionally, patients with coronary artery abnormalities received echocardiograms depending on severity of illness as part of standard of care. Patients were classified as having normal (< 2.5 SD units [z score] from the mean, normalized for body surface area), dilated (z score ≥ 2.5 to < 4), or aneurysmal (z ≥ 4; z > 10 for giant aneurysm) coronary arteries on the base of the maximal internal diameters of the right coronary artery and left anterior descending artery [16]. With cardiac involvement was meant any cardiac abnormalities (such as coronary involvement and/or pericardial effusion and/or valvular regurgitation and/or cardiac tamponade and/or arrhythmia). At first, patients were divided in those who presented cardiac involvement and those who did not. Moreover, in light of coronary impairment, children with cardiac involvement were subdivided in two groups: those with coronary artery abnormalities (CAA) and those without. Lastly, our cohort has been dividend in two further groups: children below and over 6 months of age. Time to diagnosis, cardiac involvement and CAA have been compared between the two groups.

Furthermore, complete blood count, erythrocyte sedimentation rate (ESR), C-reactive protein (CRP), alanine aminotransferase (ALT), aspartate aminotransferase (AST), sodium and albumin prior to IVIG treatment were also collected. Laboratory tests have been performed by standard methods in our clinical laboratory. The range or the minimal value of laboratory tests (i.e., ESR ≤ 20 mm/hour, CRP ≤0.29 mg/dL, ALT ≤50 UI/L, AST ≤ 35 UI/L, albumin 3,5–5 g/dl and sodium 125–135 mEq/L), detected by the standard assays, were used as appropriate.

The response to IVIG was defined as IVIG-resistance when persistent or recrudescent fever occurred between 36 h to 7 days after completion of the first IVIG infusion.

Categorical data were statistically analyzed by chi-square analysis. Continuous data were analyzed by Student t test. Adjustments were made for unequal variances using Satterthwaite’s approximation. *P* values less than 0.05 were considered statistically significant. All data were analyzed by Epi Info Statistical Software version 7.1.5.2.

According to our local regulations, ethical approval was not due for retrospective charts review.

## Results

All the demographic and clinical data are shown in Table 1, while the laboratory results are shown in Table 2.

**Table 1:** Demographic and clinical data of children with Kawasaki disease below the age of 12 months

Clinical features	Total	Complete KD (*n* = 10)	Incomplete KD (*n* = 22)
Mean age at onset	5.7 ± 2.8 months	7.4 ± 2.2 months	5.0 ± 2.8 months
Male: female ratio	20:12	4:6	16:6
Mean time to diagnosis	7 ± 3 days	6 ± 1 days	8 ± 4 days
Fever > 5 days	32/32 (100%)	10/10 (100%)	22/22 (100%)
Conjunctival injection	26/32 (81.2%)	10/10 (100%)	16/22 (72.7%)
Rash	25/32 (78.1%)	10/10 (100%)	15/22 (68.2%)
Extremity changes	18/32 (56.2%)	10/10 (100%)	8/22 (36.4%)
Mucosal changes	13/32 (40.6%)	9/10 (90%)	4/22 (18.2%)
Cervical lymphadenopathy	7/32 (21.9%)	2/10 (20%)	5/22 (22.7%)
Cardiac involvement	19/32 (59.4%)	6/10 (60%)	13/22 (59%)
Coronary abnormalities	16/32 (50%)	4/10 (40%)	12/22 (54.5%)

**Table 2:** Laboratory features of children with Kawasaki disease below the age of 12 months

Laboratory data at onset	Total	Complete KD	Incomplete KD	CAA-	CAA+
Mean ± SD Platelet count 10^3^/microL	525.9 ± 192.0	528.1 ± 137.3	524.9 ± 215.3	570.0 ± 216.8	495.7 ± 172.7
Mean ± SD Leukocyte count 10^3^/microL	16.9 ± 8.5	18.1 ± 9.2	16.4 ± 8.3	16.3 ± 8.6	17.4 ± 8.6
Mean ± SD Hemoglobin	10.6 ± 1.3	10.7 ± 1.8	10.5 ± 1.0	10.9 ± 1.1	10.4 ± 1.5
Mean ± SD ESR mm/h	60.0 ± 30.6	80.1 ± 25.7*	50.5 ± 28.6*	62.5 ± 31.4	58.1 ± 30.9
Mean ± SD CRP mg/dl	8.2 ± 6.8	9.7 ± 9.6	7.5 ± 5.0	7.7 ± 6.0	8.6 ± 7.3
Mean ± SD ALT UI/L	65.0 ± 72.3	57.6 ± 56.3	68.4 ± 79.4	56.9 ± 79.6	70.5 ± 68.5
Mean ± SD AST UI/L	74.8 ± 113.7	29.8 ± 13.2*	95.4 ± 132.7*	59.8 ± 74.5	85.2 ± 135.2

*p < 0.05

### Demographic profile

Between January 1, 2008 and December 31, 2017, 113 children have been diagnosed with KD in our hospital. Among these, 32 infants aged below 1 year at time of disease onset (28.3%) were included in the study. Nineteen patients were less than 6 months, representing 59.3% of the selected population and 16.8% of all the KD patients; 3 infants were below 3 months of age (9.4% of patients below 1 year of age and 2.6% of all patients diagnosed with KD). The youngest was a 30-day old male infant. Mean age of presentation was 5.7 ± 2.7 months. Most patients were males (M: F = 1.7:1). In terms of season of onset, 12 patients (32.5%) developed KD in December–February and 10 (31.2%) in June–August; 5/32 (15.6%) in September–November and 5/32 (15.6%) in March–May.

### Clinical features

The mean time to diagnosis from the onset (day 1 of fever) was 7 ± 3 days (range 3–22) and was longer in the incomplete forms (8 ± 4 vs 6 ± 1 days). In 3 patients the diagnosis of KD was made after day 10 of illness and two developed cardiac involvement (coronary aneurism and pericardial effusion respectively). In one of these cases, the diagnosis was even delayed till day 22 and the boy developed coronary arteries aneurisms, pericardial effusion and cardiac tamponade. Focusing on children below 6 months of age, they appeared to get a more delayed diagnosis than the older infants (7 vs 6 days), with no significant difference (*p* = 0.53). Conjunctival injection was present in 26 patients (81.2%); rash in 25 (78.1%); extremity changes in 18 (56.2%); mucosal changes in 13 (40.6%) and cervical lymphadenopathy in 7 (21.8%). Most patients (68.7%, 22 cases) had incomplete KD, in particular 7 fulfilled 3 criteria, 11 fulfilled 2 criteria and 4 fulfilled 1 criterion. With regard to cardiac involvement, it was present in about 60% in both groups. Furthermore, analyzing coronary involvement, CAA were detected in 16/32 (50%) patients, 12 of whom had an incomplete form (54.5%). Neither giant aneurisms nor arrhythmia was found. Eight (25%) patients developed a pericardial effusion. No valvular regurgitation was detected. Children below 6 months of age showed a slight greater predilection for cardiac involvement (63.2%) and coronary disease (57.9%) than older infants (53.8 and 30.8%, respectively) but without significant difference (*p* = 0.59 and 0.28, respectively).

### Laboratory data

Mean platelet count was 525.9 ± 192 10^3^/microL and 7/32 (21.8%) had a normal platelet count at onset. One patient developed thrombocytopenia (140 10^3^/microL) and one had a platelet count above 1000 10^3^/microL (1012 10^3^/microL). In terms of developing CAA, no statistically significant difference was found related to platelets count. Mean total leukocyte count was 16.9 ± 8.5 10^3^/microL. Twenty-three of 32 patients had a leukocyte count above 12.0 10^3^/microL. Sixteen of 23 (69.5%) had incomplete KD.

Mean hemoglobin level was 10.6 ± 1.3 g/dL. Six patients presented with a hemoglobin level < 10 mg/dl and 5 of them developed CAA. The median initial ESR was elevated (mean 60 ± 30 mm/h) in all patients. However, mean ESR level in the complete forms was significantly higher than in incomplete ones (80 ± 25.7 mm/h vs 50 ± 28.6 mm/h; *p* = 0.01). There was no significant difference in ESR level when comparing CAA+ and CAA- groups.

CRP was evaluated in all patients: 31 of them had raised levels, with a mean of 8.2 ± 6.8 mg/dL. Mean value in complete forms (9.7 ± 9.6 mg/dL) is higher than in incomplete ones (7.5 ± 5 mg/dL) although without any significant difference. The initial CRP level was high in a comparable percentage of those who had CAA (8.6 ± 7.3 mg/dL) and those who did not (7.7 ± 6 mg/dL).

ALT and AST mean levels were 65 ± 72.3 U/L and 74.9 ± 113.7 U/L, respectively. Focusing on AST level, it resulted much higher in incomplete forms than in complete ones (95.4 ± 132.7 UI/L vs 29.8 ± 13.2 UI/L), with a statistically significant difference (*p* = 0.03) between the two groups. On the other hand, no significant difference in transaminases level was found in patients with or without cardiac involvement.

Among the 28 patients in whom albumin levels were evaluated, 19 developed hypoalbuminemia and 15/19 (78.9%) had an incomplete form. Furthermore, 12/19 (63.1%) developed heart involvement. Six patients presented hyponatremia; 4 of them had an incomplete form. Three patients with hyponatremia developed CAA.

### Treatment

All 32 patients received standard IVIG dose (2 g/kg in single infusion) and high dose acetylsalicylic acid (ASA) at 50 mg/kg/day orally in 4 divided doses, except for 7 patients with hypertransaminasemia (AST and /or ALT > 2 DS) who skipped high dose ASA therapy for its potential hepatotoxicity. After children have been afebrile for 48 to 72 h, low antithrombotic ASA was always administered, irrespective of transaminase levels. Six children (19%), of whom 4 below 6 months, needed a second dose of IVIG and all of them presented CAA. One child required a third dose of IVIG and then additional treatment with anakinra (2 mg/kg). All patients healed from heart involvement except for 2, both below 6 months of age. Most of them recovered within 1 year, in two cases cardiac normalization was obtained within 5 years with no long-term sequelae.

## Discussion

There is paucity of data on the clinical presentation of KD in infancy and none from Italy, so far. KD below 1 year of age can be very challenging to diagnose because of unusual clinical presentations with a majority of incomplete forms and paucity of clinical signs [10, 17, 18]. It is well known that infants with KD are more likely to have cardiac involvement than older children [1–3, 10, 17, 18]. Whether the increased rate of coronary complications is exclusively due to the delayed diagnoses, and consequently to a delayed treatment, is still unclear.

Among all our patients with KD the percentage of infants, aged below 1 year (28.3%), was higher than reported in a previous study (17.5%) [17]. Considering patients younger than 6 months, they were 19 (16.8%) much more than reported by Park et al. (7.7%) [19] and by Singh et al. (3.6%) [9]. Eventually, our percentage of infants under 3-month-old (2.6%) was higher than 1.6% reported in previous studies [8, 20].

In our cohort the mean time to diagnosis was 7 days, and it was comprehensibly longer for incomplete forms. In 3 patients the diagnosis of KD was made after day 10 of illness and in one of them it was even delayed till day 22. This boy developed coronary arteries aneurisms, pericardial effusion and cardiac tamponade. Children below 6 months are reported to receive more often a late diagnosis [12]. Therefore, time to diagnosis was compared in patients aged below and over 6 months and the diagnosis resulted slightly delayed in the youngest (7 vs 6 days).

Accordingly, the 3 patients diagnosed beyond 10 days, were under 6 months of age. Thus, pediatricians need to have a high index of suspicion of KD evaluating infants, especially under 6 months, with unexplained fever for more than 5 days, especially if unresponsive to antibiotics.

As reported in literature, in our cohort the most common clinical features were conjunctival injection, rash, and extremity changes [1]. In contrast, in our cases mucosal changes were found only in 40.6%, and lymphadenopathy was detected in 21.9%. The latter finding agrees with two previous studies, reporting lymphadenopathy in 17 and 5.7% of KD infants younger than 6 months, respectively [9, 16]. Indeed, lymphadenopathy is already known as the least common of all manifestations included in classical criteria. Rather, mucosal changes that are described in literature as a usual sign, ranging from 64 to 96.5% [9, 21], were uncommonly found in our cohort. In particular, in our infants with incomplete KD, mucosal changes resulted to be the least common clinical feature (18.2%).

In previously reported series, incomplete KD represents 15–20% of cases, being more frequent in children below 12 months of age [1, 4]. In our cohort the majority of patients had incomplete KD (68.7%), which results much more common than reported by Singh et al., who found incomplete form in 35% of infants below 6 months, and in 12% of the overall KD patients [9].

Infants with KD are likely to have a more severe disease and higher risk of cardiac involvement. In our cohort cardiac involvement was present in about 60% in both complete and incomplete groups, and CAA were detected in 50% of patients, most of them had an incomplete form. Both total cardiac involvement (60%) and CAA (50%) resulted higher among our patients than previously reported (40 and 25%, respectively) [3]. Shulman et al. described 36 KD patients, who were less than l year of age during the pre-IVIG era and found that CAA developed in 31% compared with 18% in those who were 1 to 2 years of age and 10% who were more than 2 years of age [22]. Also, in the pre-IVIG era, Burns et al. reported that CAA developed in 6 out of 8 patients with KD aging less than 6 months of age [11], and Takahashi et al. reported a higher prevalence of CAA in children less than 1 year of age (39%) than in older children (13%) [23].

Cardiac involvement rate was 63.2% in children < 6 months (vs 59.4% in the older), with a higher rate of CAA (57.9% vs 50.0%), but without statistically significant difference.

As suggested in previous studies [9, 12], these findings observe that in early infancy KD is associated with an increased proportion of cardiac involvement. Delayed diagnosis and consequent delayed treatment could be responsible for the higher rate of cardiac complications.

However, it is still an ongoing debate whether the higher risk of developing CAA is solely due to the delayed diagnosis in incomplete forms or other factors might be involved. Conversely to the literature findings [24, 25], none of our patients developed arrhythmia or valvular insufficiency.

As already observed [10], in our patients’ laboratory findings were nonspecific neither diagnostic, and generally did not predict CAA development. Although most of our patients had high platelets level, thrombocytosis was not related to a higher risk of CAA development. Only one patient had thrombocytopenia and he developed CAA. Indeed, thrombocytopenia is already known as risk factor for cardiac involvement in KD [26].

In our serie, moderate up to marked leukocytosis was found in 2/3 of our patients. Nevertheless, in those with normal white blood cells count, cardiac involvement was equally present, confirming that lack of leukocytosis did not predict an uncomplicated course of illness.

ESR and CRP values were all higher in complete KD forms than in incomplete ones; among these parameters ESR value was significantly higher in the complete forms. This result confirms that incomplete KD, with relatively low inflammation markers, is ambiguous not only in terms of clinical features, but also of laboratory findings, leading to a delayed diagnosis. Furthermore, ESR, CRP and platelet levels were high in a comparable percentage of those with CAA and those without, thus they are not predictive of coronary disease, as already seen [10].

To date, AST level has not been reported to be significantly higher in incomplete forms. We gathered from our cohort a much higher AST level in incomplete forms than in complete ones, with a statistically significant difference between the two groups. This finding is quite impressive, considering that no statistically significant laboratory data has been found before to characterize incomplete KD. More studies are needed to confirm whether AST level might be included in KD criteria to detect incomplete forms, but in real-life practice a high AST level might be very useful when facing young infants with unexplained fever lasting more than 5 days, who no fulfilled KD criteria, whenever clinicians must weigh the trade-offs between a prompt IVIG therapy and the risk of missing other diagnosis.

In contrast to this finding, no significant AST level difference has been found between patients with or without cardiac involvement. Thus, AST level cannot be considered a possible biomarker of cardiac damage in KD, despite the recognition of hepatic involvement in association with other forms of myocarditis has already been described [27].

Moreover, although not statistically significant, low albumin levels were reported in higher percentage of patients with incomplete KD, as additional potential sign of liver involvement in this KD form. Liver impairment might be helpful as additional clue in the challenging diagnosis of infants with incomplete KD.

A further aid for this difficult diagnosis could come from genetic assessment. Recently, evidence for a genetic component for KD susceptibility has been described [3]. Variants in the transforming growth factor (TGF)-β signaling pathway (TGFβ2, TGFβR2, and SMAD3) genes were associated with increased risk of aneurysm formation in Caucasian patients. Further works in this area could be helpful to detect children at high risk of cardiac involvement especially below 1 year of age.

In our cohort all patients received IVIG treatment. Six children, all of them with CAA, required a second dose of IVIG 24 h thereafter: four of them were below 6 months. One child (4-month-old) presenting with incomplete form had resistant KD and required a third dose of IVIG and then additional treatment with anakinra (2 mg/kg). Indeed, according to earlier studies [19, 28], age below 6-month has been reported to be an important variable in predicting IVIG resistance. The percentage of IVIG resistance among our cohort of children was 19%, higher than reported by Egami et al. (15%) describing patients of the same age [29]. This discrepancy was even more glaring when compared to the overall IVIG resistance (10%) [29, 30]. To conclude, our results highlight a higher resistance to treatment not only in children below 6 months of age as previously reported [19, 28], but also in the ones below 1 year of age.

There is no consensus regarding optimal adjunctive therapeutics for KD refractory to intravenous immunoglobulins. Patients at high risk of coronary artery aneurysms development may benefit from adjunctive therapy. Our 4-month-old boy has been successfully treated with anakinra. Our choice was based on some evidence that anakinra, used late in the disease course, leads to a rapid and sustained improvement in clinical and biological inflammation [31]. Indeed, recently the importance of IL-1 signaling pathway has been highlighted in patients with KD [32–34].

Furthermore, we decided not to treat patients who presented hypertransaminasemia with high ASA doses to avoid a potential hepatotoxicity. This choice did not affect the cardiac outcome in our cohort as already stated by literature. In fact, although ASA has important anti-inflammatory activity (at high doses) and antiplatelet activity (at low doses), it does not appear to lower the frequency of development of coronary abnormalities [35, 36].

However, the sample size limitation would be advocated. Since our cohort encompasses only 32 patients, the increased likelihood of a Type II error, skewing the results and decreasing the power of the study, could be occur.

## Conclusions

In conclusion, KD clinical diagnosis below 1 year of age, and even more below 6 months, can be very challenging since patients may not have classic signs and symptoms, and individual manifestations may be subtle. Therefore, in young infants with unexplained fever lasting more than 5 days, a clinical possibility of KD must be considered and appropriate investigations performed. In this clinical setting, liver function test evaluation might drive the diagnosis. In fact, timely diagnosis and institution of treatment with IVIG and aspirin is required to minimize cardiac sequelae and long-term morbidity. To our knowledge this is the first study involving an Italian cohort of patients with KD below 12 months. Further studies are needed to define specific diagnostic criteria for this particular group of age, even if the final diagnosis in doubtful cases still relies on expert opinion.

## Data Availability

The datasets used during the current study are available from the corresponding author on reasonable request.

## Abbreviations

AHA: American Heart Association
ALT: Alanine aminotransferase
ASA: Acetylsalicylic acid
AST: Aspartate aminotransferase
CAA: Coronary artery abnormalities
CRP: C-reactive protei
ESR: Erythrocyte sedimentation rate
IVIG: Intravenous immunoglobulin
KD: Kawasaki disease
LAD: Left anterior descending artery
RCA: Right coronary artery
